# Increasing Rates of Brain Tumours in the Swedish National Inpatient Register and the Causes of Death Register

**DOI:** 10.3390/ijerph120403793

**Published:** 2015-04-03

**Authors:** Lennart Hardell, Michael Carlberg

**Affiliations:** Department of Oncology, Faculty of Medicine and Health, Örebro University, SE-701 82 Örebro, Sweden; E-Mail: michael.carlberg@regionorebrolan.se

**Keywords:** mobile phone, cordless phone, brain tumour incidence, Swedish National Inpatient Register, Causes of Death Register, cancer register, radiofrequency fields

## Abstract

Radiofrequency emissions in the frequency range 30 kHz–300 GHz were evaluated to be Group 2B, *i.e.*, “possibly”, carcinogenic to humans by the International Agency for Research on Cancer (IARC) at WHO in May 2011. The Swedish Cancer Register has not shown increasing incidence of brain tumours in recent years and has been used to dismiss epidemiological evidence on a risk. In this study we used the Swedish National Inpatient Register (IPR) and Causes of Death Register (CDR) to further study the incidence comparing with the Cancer Register data for the time period 1998–2013 using joinpoint regression analysis. In the IPR we found a joinpoint in 2007 with Annual Percentage Change (APC) +4.25%, 95% CI +1.98, +6.57% during 2007–2013 for tumours of unknown type in the brain or CNS. In the CDR joinpoint regression found one joinpoint in 2008 with APC during 2008–2013 +22.60%, 95% CI +9.68, +37.03%. These tumour diagnoses would be based on clinical examination, mainly CT and/or MRI, but without histopathology or cytology. No statistically significant increasing incidence was found in the Swedish Cancer Register during these years. We postulate that a large part of brain tumours of unknown type are never reported to the Cancer Register. Furthermore, the frequency of diagnosis based on autopsy has declined substantially due to a general decline of autopsies in Sweden adding further to missing cases. We conclude that the Swedish Cancer Register is not reliable to be used to dismiss results in epidemiological studies on the use of wireless phones and brain tumour risk.

## 1. Introduction

### 1.1. Background

There has been a rapid increase in the use of both mobile and cordless phones during the last two decades. Worldwide, an estimate of 6.9 billion mobile phone subscriptions were reported at the end of 2014 by the International Telecommunication Union [1]. 

The Nordic countries were among the first in the world to widely adopt wireless telecommunications technology. Analogue phones (Nordic Mobile Telephone System; NMT) were introduced in the 1980s using both 450 (1981 to 2007) and 900 (1986 to 2000) Megahertz (MHz) frequencies. The digital system (Global System for Mobile Communication; GSM) using two bands, 900 and 1800 MHz, started to operate in 1991. The third generation of mobile phones, 3G or Universal Mobile Telecommunication System (UMTS), using 1900/2100 MHz was introduced in Sweden in 2003 and dominates the market now. The fourth generation (4G; Long term evolution; LTE) started to operate first in the world in December 2009 in Norway (Oslo) and Sweden (Stockholm).

Desktop cordless phones have been used in Sweden since 1988, first using analogue 800–900 MHz frequencies, but since early 1990s using a digital 1900 MHz system (Digital Enhanced Cordless Telecommunications; DECT).

The real increase in the use and exposure to radiofrequency electromagnetic fields (RF-EMF) from wireless phones (mobile phones and cordless phones) in most countries has occurred since the 1990s. The anatomical distribution to the human body of RF emissions depends on e.g., if the wireless phone is handheld or not, type of tissue and age [2,3,4]. The user may also be exposed to extremely low frequency (ELF)-EMF from the battery [5,6].

The brain is the primary target organ for exposure to EMF during the use of the handheld phone. This has given concern of an increased risk for brain tumours. The carcinogenic effect of RF-EMF on humans was evaluated at a meeting during 24–31 May 2011 at the International Agency for Research on Cancer (IARC) at WHO in Lyon, France. The Working Group categorised RF-EMF from mobile phones, and from other devices that emit similar non-ionising electromagnetic fields in the frequency range 30 kHz–300 GHz, as Group 2B, *i.e.*, “possibly”, carcinogenic to humans [7,8].

Case-control studies on brain tumour risk provided supportive results on positive associations between two types of brain tumours; glioma and acoustic neuroma, and exposure to RF-EMF from wireless phones [9,10,11,12,13,14]. The results for meningioma were less consistent for an association. Recently a decreased survival of patients with glioblastoma multiforme associated with long-term use of wireless phones was reported [15]. Another study reported mobile phone use for ≥3 h a day to be associated with a consistent pattern of increased risk for the mutant type of *p53* gene in the peripheral zone of the glioblastoma, and that this increase was statistically significant correlated with shorter overall survival time [16]. 

### 1.2. Incidence

It has been suggested that overall incidence data on brain tumours for countries may be used to qualify or disqualify the association between mobile phones and brain tumours observed in case-control studies. During recent years such opinions have been published by different study groups. However, it must be stressed that descriptive epidemiology with no individual exposure data is of less value than results in analytical epidemiology such as case-control studies [17].

Aydin *et al.* [18] made a case-control study on mobile phone use among children and adolescents in Sweden, Denmark, Norway and Switzerland. In spite of elevated odds ratios (ORs) incidence data from the Swedish Cancer Register during 1990–2008 for the age group 5–19 years were used to dismiss the significance of the results for all countries: “*our evaluation of time trends of brain tumor incidence in Sweden and altogether provide little support to the view that mobile phone use increases the risk of brain tumors*”. The many shortcomings in the analysis including hypothetical incidence rate trends was commented by us elsewhere [19].

Deltour *et al.* [20] reported increasing glioma incidence rates in Denmark, Finland, Norway, and Sweden for the time period 1979–2008. Annual percentage change (APC) increased for men with +0.4%, 95% confidence interval (CI) +0.1, +0.6% and for women with +0.3%, 95% CI +0.1, +0.5%. In simulations using various risk estimates and induction times for mobile phone use, it was concluded that: “*Several of the risk increases seen in case-control studies appear to be incompatible with the observed lack of incidence rate increase in middle-aged men*”*.* Unfortunately no data were given for subtypes of glioma and anatomical sites of the tumours, which would certainly have been informative. The authors did not consider these and other limitations such as the quality of cancer register data when they concluded that “*Our data indicate that, so far, no risk associated with mobile phone use has manifested in adult glioma incidence trends...many increased or decreased risks reported in case-control studies are implausible, implying that biases and errors in the self-reported use of mobile phone have likely distorted the findings*”.

The age-standardized incidence of brain tumours increased in Denmark with +41.2% among men and +46.1% among women during 2003–2012 [21]. A news release based on the Danish Cancer Register stated that during the last 10 years there has been an almost 2-fold increase in the incidence of the most malignant glioma type, glioblastoma multiforme [22]. So far these incidence data are not generally available.

The Scientific Committee on Emerging and Newly Identified Health Risks (SCENIHR) at EU published on 12 December 2013 a “*Preliminary opinion on potential health effects of exposure to electromagnetic fields (EMF)*” [23]. The committee’s conclusion was that “*Based on the most recent cohort and incidence time trend studies, it appears that the evidence for an increased risk of glioma became weaker while the possibility of an association of RF EMF exposure with acoustic neuroma remains open*”. The conclusion in the final report was that “*The results of cohort and incidence time trend studies do not support an increased risk for glioma while the possibility of an association with acoustic neuroma remains open*” (Dated 20 January 2015) [24].

In September, 2014 WHO published the draft: *Radio Frequency fields: Environmental Health Criteria Monograph* [25]. The results by Deltour *et al.* [20] on brain tumour incidence in the Nordic countries were cited without further analysis of the findings: “*if the risk increases that were reported in a few case-control studies to be associated with mobile phone use were real, they would have resulted in a detectable increase in the glioma incidence in the Nordic countries*”*.*

### 1.3. Aim of the Study

It is obvious that descriptive data from the Swedish Cancer Register on brain tumour incidence are increasingly used to dismiss results in analytical epidemiology on the association between brain tumours and use of mobile and cordless phones. An increasing number of patients with brain tumour of “unknown type” based on patient register data was recently reported [26]. Thus it is pertinent to analyse the relevance of the Swedish Register data. This was a register based study using official data without any personal identification. Thus approval by the ethical committee was not necessary.

## 2. Material and Methods

### 2.1. Study Design

In this study official registers in Sweden without any information on personal identification were used. Data were retrieved from the Swedish National Inpatient Register (IPR) available online [27]. This register was established in 1964. It is mandatory to report the patients to the register and it has complete national coverage since 1987 [28]. Currently more than 99% of hospital discharges are registered. Data were analysed for the time period 1998–2013. We report number of patients per 100,000 inhabitants. Age-standardized rates are not available in the register.

The Swedish Causes of Death Register (CDR) was established in 1961 and includes currently all deaths (99.5%) among Swedish residents and is available online [29]. Also from that register data for the same time period as in IPR, 1998–2013, were assessed. Age-standardized death rates per 100,000 inhabitants according to the Swedish population in 2000 were used.

The following codes for brain tumour diagnosis were analysed in IPR and CDR according to ICD-10 code: D32 = Benign meningeal tumours (CNS); D33 = Benign tumour in the brain or CNS; D42 = Tumour of unknown type in the meninges (CNS); D43 = Tumour of unknown type in the brain or CNS; C71 = malignant brain tumour. Online data was available for both genders combined and separately.

The National Board of Health and Welfare administers the Swedish Cancer Register that started in 1958. It is compulsory for all health care providers to report new diagnostic cancer cases to the register. The basis for diagnosis can be clinical examination, histology/cytology, surgery, autopsy, or other examinations such as CT/MRI or laboratory investigations. Incidence per 100,000 person-years age-adjusted according to the World population was analysed for the ICD-7 code 193.0 = brain tumour based on data in the Swedish Cancer Register for the time period 1998–2013 [30]. It should be noted that it includes both malignant and benign brain tumours in contrast to ICD-10 code C71 (only malignant). Online data was available for men and women separately (*i.e.*, not combined).

Frequency of autopsy as the basis for diagnosis of nervous system tumours (ICD-7 = 193; data not available for 193.0) and verification by histology or cytology were based on yearly publications of Cancer Incidence in Sweden available at the National Board of Health and Welfare for the time period 1980–2013.

### 2.2. Statistical Methods

The NCI Joinpoint Regression Analysis program, version 4.1.1.1 was used to examine trends in age-standardized death rates, number of patients per 100,000 in inpatient care and age-standardized incidence by fitting a model of 0–3 joinpoints using settings in default mode [31]. When joinpoints were detected APC and 95% CIs were calculated for each linear segment. Average annual percentage changes (AAPC) were also calculated for the whole time period using the average of the APCs weighted by the length of the segment.

Restricted cubic splines were used to display the relationship between latency (time period from first use of mobile phone to diagnosis of brain tumour) of mobile phones with glioma in our case-control studies [32]. Adjustment was made for the matching variables gender, age (as a continuous variable), year of diagnosis, and in addition socio-economic index (SEI) divided into four categories (blue-collar worker, white-collar worker, self-employed, unemployed). For further details see Hardell, Carlberg [32].

## 3. Results

### 3.1. Inpatient Register (IPR) and Causes of Death Register (CDR)

#### 3.1.1. D32 Benign Meningeal Tumours (CNS)

According to the IPR, AAPC increased slightly during 1998–2013, although not statistically significant, see Table 1. No joinpoint was detected. In the CDR, AAPC decreased statistically significant during 1998–2003 with −2.96%, 95% CI −4.82, −1.05% for both genders combined, Table 2. One joinpoint was detected, 2007, in men (2007–2013: APC −11.74%, 95% CI −19.84, −2.82%). A decreasing AAPC was also found in women, but no joinpoint was detected.

#### 3.1.2. D33 Benign Tumour in the Brain or CNS

A slight decrease of AAPC was seen both in men and women in the IPR. The result was not statistically significant and no joinpoint was detected, see Table 1. The results in the CDR were based on only 82 patients and thus less reliable although with decreasing AAPC for both genders combined for the whole time period, Table 2.

#### 3.1.3. D42 Tumour of Unknown Type in the Meninges (CNS)

A statistically significant decreasing AAPC in IPR was found for both genders during 1998–2003, Table 1. A joinpoint was found in 2003 for men. The results were based on rather low numbers. Only 21 cases were reported to CDR so analysis of the data could not be done.

**Table 1:** Joinpoint regression analysis of trends in number of patients per 100,000 in the Swedish National Inpatient Register (IPR) 1998–2013 [27].

ICD-10	Joinpoint Location	APC 1 (95% CI)	APC 2 (95% CI)	AAPC (95% CI)
**D32**				
All (*n* = 10,962)	No joinpoint detected	-	-	+0.49 (−0.06, +1.04)
-Men (*n* = 3183)	No joinpoint detected	-	-	+0.88 (+0.12, +1.64)
-Women (*n* = 7779)	No joinpoint detected	-	-	+0.37 (−0.32, +1.06)
**D33**				
All (*n* = 6925)	No joinpoint detected	-	-	−0.43 (−0.97, +0.11)
-Men (*n* = 3141)	No joinpoint detected	-	-	−0.63 (−1.40, +0.14)
-Women (*n* = 3784)	No joinpoint detected	-	-	−0.26 (−0.88, +0.37)
**D42**				
All (*n* = 1093)	No joinpoint detected	-	-	−2.10 (−3.60, −0.58)
-Men (*n* = 433)	2003	+6.41 (−3.28, +17.07)	−6.71 (−9.76, −3.56)	−2.53 (−5.84, +0.90)
-Women (*n* = 660)	No joinpoint detected	-	-	−1.26 (−2.76, +0.26)
**D43**				
All (*n* = 13,013)	2007	+0.17 (−1.01, +1.37)	+4.25 (+1.98, +6.57)	+1.78 (+0.76, +2.81)
-Men (*n* = 6840)	2007	+0.13 (−1.62, +1.90)	+4.95 (+1.59, +8.42)	+2.03 (+0.52, +3.56)
-Women (*n* = 6173)	2008	+0.36 (−0.41, +1.14)	+4.08 (+1.80, +6.41)	+1.58 (+0.77, +2.40)

ICD-10: D32 = Benign meningeal tumours (CNS); D33 = Benign tumour in the brain or CNS; D42 = Tumour of unknown type in the meninges (CNS); D43 = Tumour of unknown type in the brain or CNS. APC = Annual Percentage Change (APC 1 = time from 1998 to joinpoint; APC 2 = time from joinpoint to 2013); AAPC = Average Annual Percentage Change.

**Table 2:** Joinpoint regression analysis of trends in age-standardized death rates per 100,000 in the Swedish Causes of Death Register (CDR) 1998–2013 [29].

ICD-10	Joinpoint Location	APC 1 (95% CI)	APC 2 (95% CI)	AAPC (95% CI)
**D32**				
All (*n* = 845)	No joinpoint detected	-	-	−2.96 (−4.83, −1.05)
-Men (*n* = 282)	2007	+3.25 (−1.98, +8.76)	−11.74 (−19.84, −2.82)	−3.03 (−7.21, +1.35)
-Women (*n* = 563)	No joinpoint detected	-	-	−3.26 (−5.39, −1.09)
**D33**				
All * (*n* = 82)	No joinpoint detected	-	-	−7.01 (−12.72, −0.92)
**D43**				
All (*n* = 2374)	2008	−7.35 (−10.87, −3.68)	+22.60 (+9.68, +37.03)	+1.72 (−2.29, +5.90)
-Men (*n* = 1166)	2008	−8.75 (−12.69, −4.64)	+23.98 (+9.24, +40.71)	+1.06 (−3.46, +5.80)
-Women (*n* = 1208)	2008	−5.95 (−10.58, −1.07)	+19.91 (+3.70, +38.65)	+1.99 (−3.23, +7.48)

ICD-10: D32 = Benign meningeal tumours (CNS); D33 = Benign tumour in the brain or CNS; D43 = Tumour of unknown type in the brain or CNS. APC = Annual Percentage Change (APC 1 = time from 1998 to joinpoint; APC 2 = time from joinpoint to 2013); AAPC = Average Annual Percentage Change. * Not possible to analyze by gender since there were no deaths from this diagnosis some years.

#### 3.1.4. D43 Tumour of Unknown Type in the Brain or CNS

**Figure 1:**
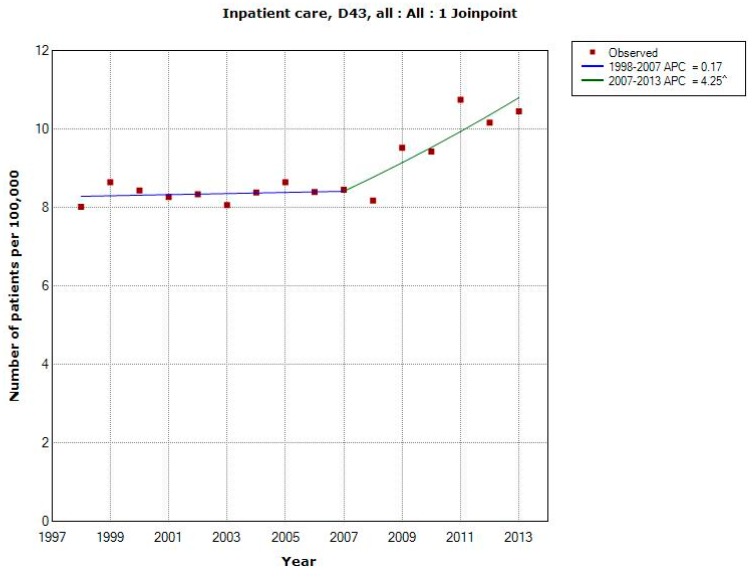
Joinpoint regression analysis of number of patients per 100,000 inhabitants according to the Swedish National Inpatient Register for both genders combined, all ages during 1998–2013 diagnosed with D43 = tumour of unknown type in the brain or CNS [27]. ^ Indicates statistically significant trend.

Figure 1 shows number of patients per 100,000 inhabitants according to the IPR for both genders combined, all ages during 1998–2013. One joinpoint was detected in 2007 (1998–2007: APC +0.17%, 95% CI −1.01, +1.37%; 2007–2013: APC +4.25%, 95% CI +1.98, +6.57%). AAPC for the whole time period was +1.78%, 95% CI +0.76, +2.81%, Table 1. The trends were similar in men and women although for women the joinpoint was detected in 2008.

**Figure 2:**
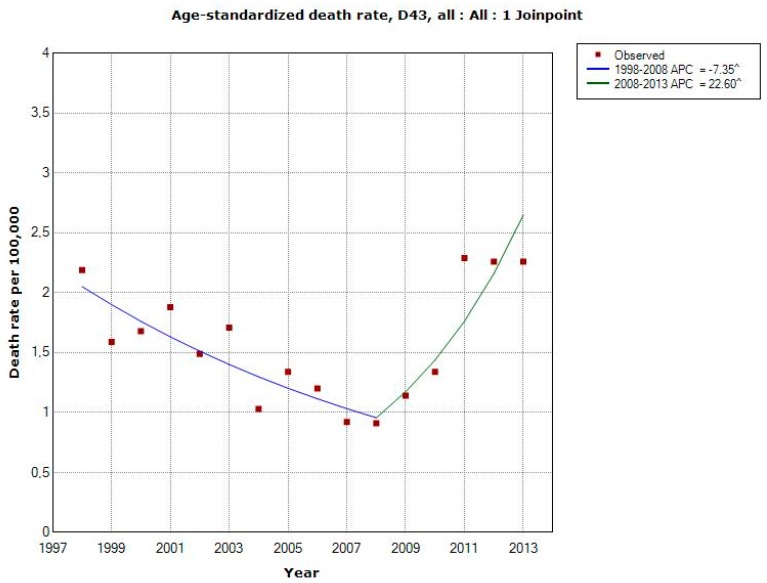
Joinpoint regression analysis of age-standardized death rates per 100,000 inhabitants according to the Swedish Causes of Death Register for both genders combined, all ages during 1998–2013 diagnosed with D43 = tumour of unknown type in the brain or CNS [29]. ^ Indicates statistically significant trend.

Figure 2 shows death rates of patients diagnosed with D43 during 1998–2013 according to the CDR. Joinpoint regression found one joinpoint in 2008 showing a statistically significant decrease for both genders combined during 1998–2008 (APC −7.35%, 95% CI −10.87, −3.68%) and a statistically significant increase during 2008–2013 (APC +22.60%, 95% CI +9.68, +37.03%), Table 2. The latter increase was seen in both genders (men APC +23.98%, 95% CI +9.24, +40.71% 2008–2013, women APC +19.91%, 95% CI +3.70, +38.65% 2008–2013). For the total period AAPC increased somewhat for both genders combined, +1.72%, 95% CI = −2.29, +5.90%. 

#### 3.1.5. C71 = Malignant Brain Tumours

In IPR AAPC for malignant brain tumours was calculated to −0.31%, 95% CI −0.97, +0.36% (men and women combined) for the time period 1998–2013. Two joinpoints were detected, 2001 and 2006 (1998–2001: APC −3.60%, 95% CI −5.96, −1.17%; 2001–2006: APC +2.11%, 95% CI +0.52, +3.72%, and 2006–2013: APC −0.58%, 95% CI −1.23, +0.08%, receptively). In the CDR the corresponding result was −1.19%, 95% CI −2.37, −0.01%. One joinpoint was found in 2008 (1998–2008: APC +1.17%, 95% CI +0.01, +2.34%, and 2008–2013: APC −5.76%, 95% CI −8.82, −2.60%, respectively), data not in table.

### 3.2. The Swedish Cancer Register

#### 3.2.1. ICD-7 Code 193.0 = Brain Tumours (Including Brain, Meninges, CNS Nerves)

Trends in age-standardized incidence rates per 100,000 were calculated. For the time period 1998–2013 AAPC was calculated to +0.06%, 95% CI −0.57, +0.69% in men, and to +0.17%, 95% CI −0.60, +0.95% in women. No joinpoint was detected, data not in table.

Sweden consists of 21 counties. We compared rates of brain tumours in the two counties with largest population. For the time period 1998–2013 APC was calculated in the Stockholm County to −0.41%, 95% CI −2.38, +1.59% and in the Västra Götaland County to +1.72%, 95% CI −0.53, +4.02 in men. In women APC was −0.81%, 95% CI −2.39, +0.80% (Stockholm County), and +2.15%, 95% CI −1.22, +5.64% (Västra Götaland County), data not in table.

#### 3.2.2. Autopsy, Histology and Cytology

**Figure 3:**
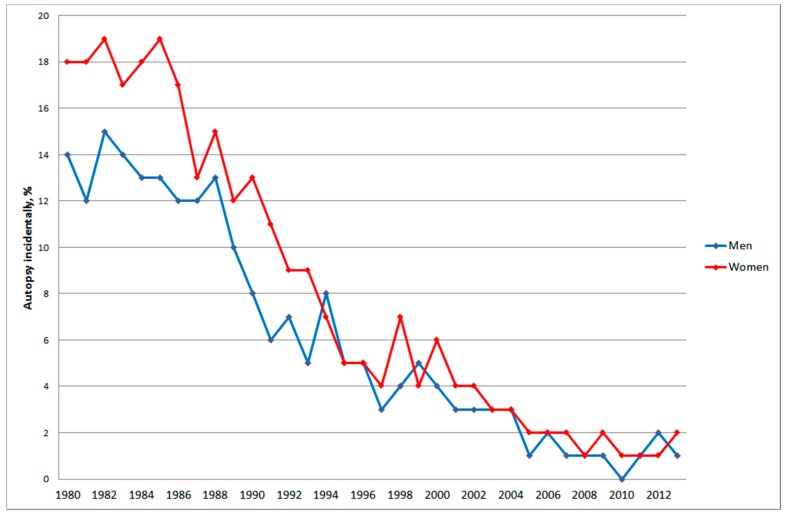
Frequency of nervous system tumours (ICD-7 code 193) based on autopsy for all ages during 1980–2013 according to the Swedish Cancer Register.

In 1980 almost 20% of nervous system tumours (ICD-7 code 193) in women and about 15% in men were based on autopsy, Figure 3. 

**Figure 4:**
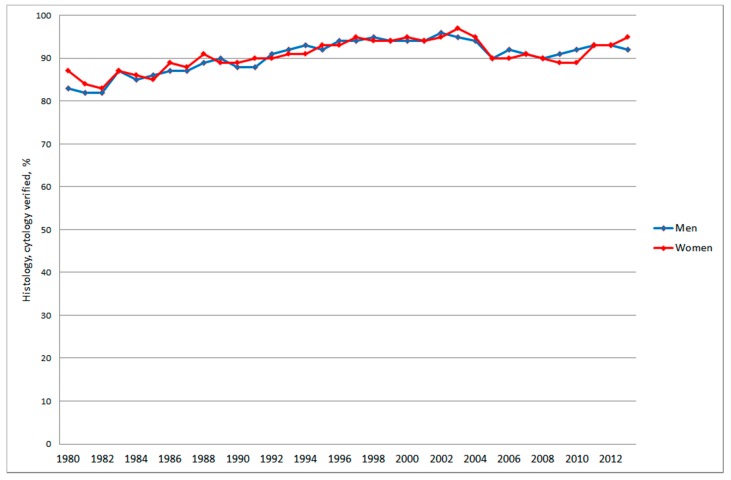
Frequency of nervous system tumours (ICD-7 code 193) confirmed with histology or cytology for all ages during 1980–2013 according to the Swedish Cancer Register.

This frequency declined dramatically to only a few percentages during the last decade, in fact only 1% to 2% since 2005, in 2010 = 0% in men. During the same time period the frequency of confirmation of the diagnosis with histology and/or cytology increased somewhat to about 90% or more since early 1990’s although with some variation over the years, Figure 4.

### 3.3. Mobile Phone Communication

**Figure 5:**
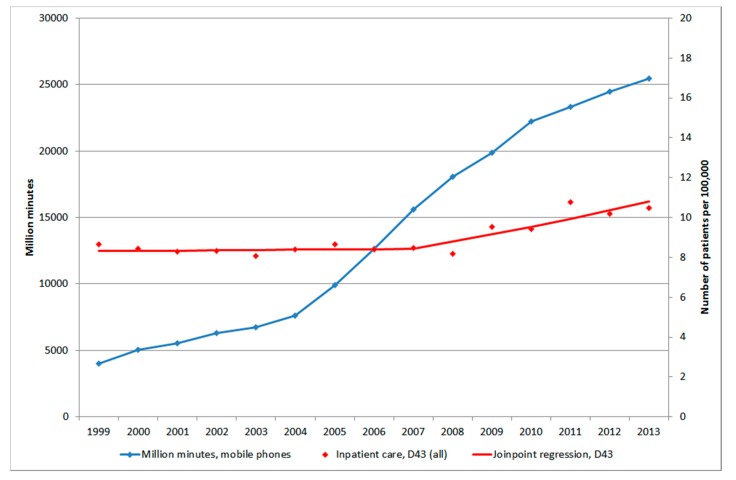
Number of out-going mobile phone minutes in millions during 1999–2013 [33] and joinpoint regression analysis of number of patients per 100,000 inhabitants according to the Swedish National Inpatient Register for all ages during 1999–2013 diagnosed with D43 = tumour of unknown type in the brain or CNS [27].

Figure 5 shows number of total minutes of out-going calls (in million minutes) during 1999–2013, data not available for 1998 [33]. 

**Figure 6:**
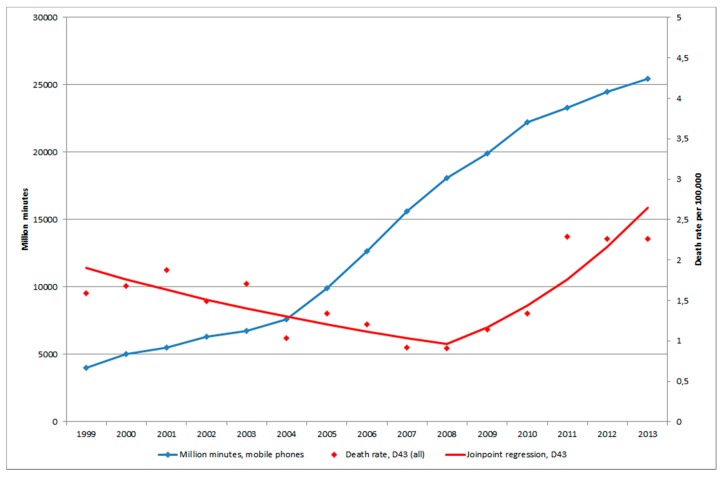
Number of out-going mobile phone minutes in millions during 1999–2013 [33] and joinpoint regression analysis of age-standardized death rates per 100,000 inhabitants according to the Swedish Causes of Death Register for all ages during 1999–2013 diagnosed with D43 = tumour of unknown type in the brain or CNS [29].

There was an increase from 2004 due to lower price from March 2003 to the end of 2005. The cost for cheapest use was reduced by 38% for the average user [34]. The number of patients per 100,000 with brain tumour of unknown type, D43, in IPR and age-standardized rate of death rate per 100,000 in CDR started to increase a few years later than 2004, see Figure 5 and Figure 6.

### 3.4. Restricted Cubic Spline Plot

**Figure 7:**
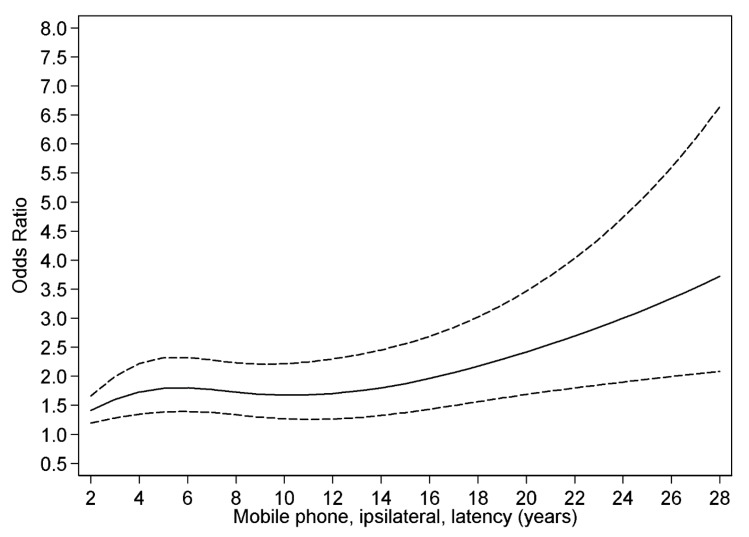
Restricted cubic spline plot of the relationship between latency of ipsilateral mobile phone use and glioma. The solid line indicates the OR estimate and the broken lines represent the 95% CI. Adjustment was made for age at diagnosis, gender, SEI-code and year for diagnosis. Population based controls were used [32].

Figure 7 shows restricted cubic spline plot of the results for glioma and latency for ipsilateral (use of mobile phone on the same side of the head as the tumour was later diagnosed) mobile phone use in our case-control study on glioma [32]. 

We found a higher OR with short latency, and after some decline an increasing risk with longer latency (non-linearity, *p* = 0.01). The results indicate both an early effect in glioma genesis (initiation), but also a late effect in carcinogenesis (promotion/progression). The increasing rates of brain tumours in Figure 5 and Figure 6 may be explained by a late effect in carcinogenesis (promotion/progression) from exposure to RF-emissions from mobile phone use.

## 4. Discussion

The main finding in this study was increasing rates of brain tumours of unknown type in the central nervous system (D43). One might speculate that these tumours represent metastases to the brain of tumours at other sites in the body. This is however unlikely since these are coded as tumours located at the original site. In the IPR the number per 100,000 patients was relatively stable until 2007 when a joinpoint was detected for men and 2008 for women. After that year the rate increased statistically significant yearly with almost 5% in men and more than 4% in women. The tumour may be calculated several times if registered during several years. It is however unlikely that this explains the clear change of the rate in Figure 1 and of course not the increasing death rate in Figure 2. Also it must be stressed that the same diagnosis is not included several times in the IPR during the same year if the person moves from one county to another, for example from a county hospital to a university hospital. All counties in Sweden are included in the same register and only unique personal id-numbers are used.

It is striking that the yearly age-standardized death rates per 100,000 inhabitants increased in the CDR from 2008 for brain tumours of unknown type (D43), the joinpoint in both men and women. The yearly statistically significant increase was 24% in men and 20% in women. For the time period 1998–2008 the rate decreased statistically significantly in both men, 9%, and in women, 6%. These results indicate that the increasing rate in the IPR is caused by inpatients with a malignant brain tumour with short survival, since the total (men and women) joinpoint shifted in 2007 in IPR, but one year later, 2008, in CDR.

**Figure 8:**
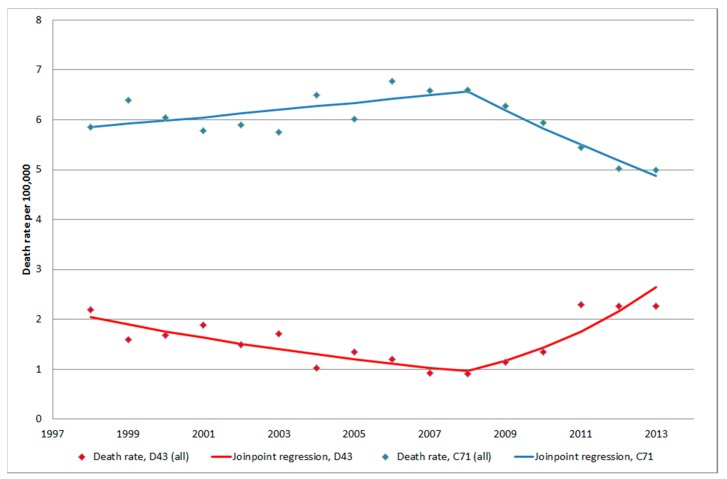
Joinpoint regression analysis of age-standardized death rates per 100,000 inhabitants according to the Swedish Causes of Death Register for both genders combined, all ages during 1998–2013 diagnosed with D43 = tumour of unknown type in the brain or CNS and C71 = malignant brain tumours [29].

**Figure 9:**
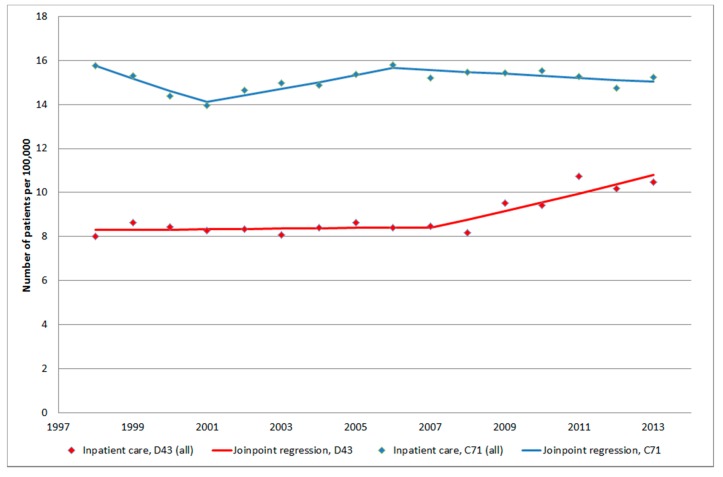
Joinpoint regression analysis of number of patients per 100,000 inhabitants according to the Swedish National Inpatient Register for both genders combined, all ages during 1998–2013 diagnosed with D43 = tumour of unknown type in the brain or CNS and C71 = malignant brain tumours [27].

It should be noted that for both tumours of unknown type (D43) and malignant brain tumours (C71) the same year, 2008, for joinpoint was detected in CDR. Thus, during 2008–2013 the increasing rate for D43 and decreasing rate for C71 might be inversely related, see Figure 8. However clearly it can be seen in Figure 9 that the statistically significant increasing rate of D43 in IPR 2007–2013 cannot be explained by the non-statistically significant decreasing rate of C71 in IPR 2006–2013. It might be speculated that the diagnosis is coded both as an unspecified brain tumour (D43) and as malignant brain tumour (C71) at the same time. This is unlikely since at the hospital discharge the diagnosis is usually based on best available data (cytology/histopathology). Furthermore, the results in Figure 9 do not support that suggestion. A patient might, however, be diagnosed with the code D43 first and at a later hospital discharge the same year with C71. This notion is not supported by the results in Figure 9; there would have been an increasing rate of C71 also if this was a common case.

In contrast to brain tumours of unknown type only a slight increase of AAPC was found in IPR during 1998–2013 for benign meningeal tumours (D32), the majority would be meningioma. The AAPC for these tumours decreased in the CDR. The reason for that is unclear but might indicate better treatment options for this patient group, other clinical routines or most probably not reported to CDR as the cause of death. The latter is supported by the low number of cases in CDR compared with IPR.

Tumours of unknown type in the meninges (D42) were rather few and AAPC decreased statistically significant in the IPR. In men a joinpoint was detected in 2003, but these results were based on low numbers. The decreasing AAPC in both genders might reflect better diagnosis and in fact improved diagnosis leading to classification as benign meningeal tumours (D32).

Benign tumours in the brain or CNS (D33) were a rather large group in the IPR. There was a slight decrease of AAPC but not statistically significant. Due to low numbers analysis of the CDR was less meaningful. This is a heterogeneous group of tumours that do not permit any firm conclusions.

All data in this study were based on official statistics in Sweden. Gender specific APC and AAPC in IPR and CDR were calculated per 100,000 persons. The data were not adjusted for age in the IPR since such information is lacking in that register. However, during a limited time period the age groups would be relatively stable in Sweden. In contrast, data in the CDR and the Swedish Cancer Register permitted calculation of trends in age-standardized rates per 100,000 subjects.

It is postulated that glioma patients would need more inpatient hospital care than patients with benign brain tumours such as meningioma. Only 1%–3% of meningioma cases have an anaplastic tumour, Grade III, with poor survival, one series reported a median survival of 2 years [35]. Atypical meningioma, Grade II, constitute 5%–7% of all cases whereas 90% are benign Grade I meningioma. Using gamma knife radiosurgery long-term survival has been reported for meningioma with 20-year actuarial rates of freedom from tumour progression of 87% [36]. Thus most meningioma cases would not need much hospital care as inpatients. 

Most of glioma cases are astrocytoma. About 5% of all astrocytoma consist of the pilocytic type (WHO grade I) that occurs mainly in children and young adults. Long-term survival, even decades, is common and malignant transformation is rare. 

Diffuse astrocytoma (WHO grade II) represents 10%–15% of all astrocytoma. The peak incidence is among young adults aged 30 to 40 years with a slight predominance in males. The mean survival after surgery is 6–8 years. The prognosis is however mainly influenced by malignant progression to glioblastoma, which has been reported to occur after 4–5 years [37,38].

Anaplastic astrocytoma (WHO grade III) is a diffuse infiltrating tumour that primarily affects adults with mean age about 40 years. It may arise from a diffuse astrocytoma, WHO grade II, or without evidence of a malignant precursor. Without surgery it has a strong tendency for progression to glioblastoma (WHO grade IV) with a mean time of about 2 years [39].

The most common primary brain tumour is glioblastoma (WHO grade IV) that accounts for about 60%–75% of all astrocytic tumours. The peak incidence is between 45 to 75 years of age. Secondary glioblastoma develops from diffuse astrocytoma, WHO grade II, or from anaplastic astrocytoma, WHO grade III. Secondary glioblastoma is less frequent than primary, less than 10%, and typically develops in younger persons, mean age 45 years. The time interval from diffuse astrocytoma grade II to glioblastoma grade IV varies between less than 1 year to more than 10 years, with mean interval 4–5 years [38,40]. Survival of patients with secondary glioblastoma has been reported to be longer (median 7.8 months), than for primary glioblastoma (median 4.7 months) [39,41].

During 1980–2013 the frequency of autopsy based diagnosis of nervous system tumours in the Swedish Cancer Register decreased from almost 20% in men and about 15% in women to 0 to 2% in both genders. This should have contributed to the statistically significant decreasing APC for brain tumours (ICD-7 code = 193.0) in men during 1980–2013 with −0.36%, 95% CI −0.58, −0.13%. In women there was a non-significant increase, APC +0.06%, 95% CI −0.20, +0.32% for the same time period.

Of all cancer diagnoses 9% in men and 6% in women were incidentally found at autopsy in 1980 *versus* 0% in both genders in 2013. There has been a general decline of the frequency of all autopsies in Sweden from about 50% in the early 1970’s to about 11% in 2013 for both men and women. In men aged 75+ years the frequency has declined from 80% since 1987 to only 6% in 2013 [42]. The lower frequency of autopsy may cause registration of the wrong cause of death in the Death Certificate. It is especially alarming that during the last years only 0–2% of CNS tumours are detected during autopsy.

In 1980 83% in men and 87% in women with nervous system tumours (ICD-7 code = 193) reported to the cancer registry were verified by histology and/or cytology. This frequency increased to about 90% or more in both genders from early 1990’s, in 2013 for men 92% and for women 95%. During our study period 1998 to 2013 this frequency has been similar during the whole time period. Using CT and MRI provides good information on type of brain tumour that can be used for clinical decisions on treatment, whereby expectancy (watchful waiting) is one option for e.g., low-grade glioma and meningioma. There may also be aggressive tumours such as glioblastoma that are diagnosed by CT and MRI, but may not be operated if the tumour has a difficult anatomical localisation, the patient is in a severe clinical condition or is old. These patients would contribute to the increasing rate in IPR, but due to the bad prognosis with short survival also the increasing rate in CDR. If all these clinically detected tumours had been reported to the Cancer Register one would expect decreasing frequency of cases with histology and/or cytology verification of the diagnosis, which in fact is not the situation in the Cancer Register. 

It should also be noted that there seems to be large regional differences in reporting to the Cancer Register, which the results from Stockholm and Västra Götaland counties show. Furthermore it should be noted that the ICD-7 code 193.0 in the Cancer Register includes both malignant and benign tumours, whereas ICD-10 code C71 in IPR and CDR represents only malignant brain tumours. Thus, these results are not comparable. No firm conclusions can be drawn regarding the decreasing AAPC for ICD-10 code C71 in IPR and CDR and these results are not comparable with ICD-7 code 193.0 in the Cancer Register including both benign and malignant brain tumours.

Quality control has shown that brain tumours are among the tumour types with large deficits in the reporting to the Cancer Register [43]. In a sample from 1998 of 202 medical records 93 cancer cases were identified that should have been reported to the Cancer Register. Of these 30% had a cancer diagnosis verified by histology (19%) or cytology (11%) whereby the pathology departments around Sweden had missed to report these cases. For 70% the cancer diagnosis was based on clinical examination only and were thus not reported to the Cancer Register by the clinical departments. Of patients with nervous system tumour the ratio of the number of persons with a tumour reported to the IPR to the number of persons reported to the Swedish Cancer Register was 48.2% for university hospitals, 121.1% (more than half not reported) for county hospitals, 4.5% for local hospitals and nursing homes, or 13.9% in total. This was in spite of reporting being mandatory and at least most pathology departments have routines for that.

A study of patients reported to the Swedish register of palliative care in 2009 as deceased due to cancer indicated that 12.5% of patients dying of cancer were not reported to the Cancer Register [44]. Radiologic investigation without a biopsy was the most common basis for diagnosis and the majority of not reported cancers were tumours in visceral organs that are easily visualized by modern imaging techniques such as CT scan but hard to reach for biopsy. Brain tumours were not part of the study but the same discussion may be applied for these tumours, especially glioblastoma multiforme. These tumours are easily diagnosed with CT/MRI but may be difficult to take a representative biopsy from or operate without a risk for neurological complications. There are limited therapeutic possibilities and short survival for these patients. It should be especially noted that most cases with nervous system tumours (ICD-7 code 193) in the Cancer Register have a diagnosis confirmed with histology or cytology as can be seen in Figure 4. The frequency has even increased since early 1990’s. That figure clearly indicates that cancer cases with only clinical diagnosis such as CT and/or MRI are underreported to the Cancer Register.

A population-based incidence study on pancreatic and biliary tract cancer for the years 1990–2009 found an overwhelming underreporting of these cancers within the Swedish Cancer Register [45]. Thus during 1990–1994 63% of pancreatic cancer cases were reported, but declined to 44% in 2005–2009. The corresponding results for biliary tract cancer were 60% and 37%, respectively. These results reflected the lack of tissue samples for histology. It was concluded that these errors may explain the reported decline of these cancer cases in the Swedish Cancer Register during recent years. 

Incidence data on brain tumours in the Swedish Cancer Registry are used to dismiss the increased risk associated with use of wireless phones in our studies [18,20]; see also SCENIHR and WHO [above]. Interestingly this study showed increasing rates of brain tumours of unknown type with joinpoint 2007 (all). This may be in accordance with our findings of increased glioma risk associated with use of wireless phones. In fact this increase is some years after increasing number of out-going mobile minutes. These data do also fit with our restricted cubic spline plot with a late effect in carcinogenesis [32], see also Appendix II in the Interphone study [12]. Tumour promotion by RF-EMF exposure was reported in 2010 in a study on mice [46]. These findings were recently replicated [47] and add to the relevance of our results. An even more increasing rate of brain tumours in the future may be predicted based on these data. In contrast the data do not show a statistically significant increase of the rate of meningioma, a tumour type that has no consistent association with use of wireless phones [8,48]. Only ionizing radiation is since before an established risk factor for brain tumours. However there are no data indicating increasing such exposure in the Swedish population that may explain our results.

## 5. Conclusions

In summary this study shows that the Swedish Cancer Register is not reliable to be used to dismiss results in epidemiological studies on the use of wireless phones and brain tumour risk and should not be used as reference for such statements. In fact both the IPR and CDR indicate a considerable underreporting to the Cancer Register during recent years. Record linkage between IPR, CDR and the Swedish Cancer Register should be the next step to further elucidate this underreporting.
